# Gestational Caloric Restriction Alters Adipose Tissue Methylome and Offspring’s Metabolic Profile in a Swine Model

**DOI:** 10.3390/ijms25021128

**Published:** 2024-01-17

**Authors:** Berta Mas-Parés, Sílvia Xargay-Torrent, Gemma Carreras-Badosa, Ariadna Gómez-Vilarrubla, Maria Niubó-Pallàs, Joan Tibau, Josep Reixach, Anna Prats-Puig, Francis de Zegher, Lourdes Ibañez, Judit Bassols, Abel López-Bermejo

**Affiliations:** 1 Obesity and Cardiovascular Risk in Pediatrics, Girona Biomedical Research Institute (IDIBGI), 17190 Salt, Spain; bmas@idibgi.org (B.M.-P.); alopezbermejo@idibgi.org (A.L.-B.); 2 Materno-Fetal Metabolic Research, Girona Biomedical Research Institute (IDIBGI), 17190 Salt, Spain; 3 Benestar Animal, Institut de Recerca i Tecnología Agroalimentàries (IRTA), 17121 Monells, Spain; joan.tibau@irta.cat; 4 Selecció Batallé, 17421 Riudarenes, Spain; 5 Department of Physical Therapy, EUSES, University of Girona, 17190 Salt, Spain; aprats@euses.cat; 6 Department of Development and Regeneration, University of Leuven, 3000 Leuven, Belgium; 7 Endocrinology, Fundació Sant Joan de Déu, University of Barcelona, 08950 Esplugues de Llobregat, Spain; 8 Centro de Investigación Biomédica en Red de Diabetes y Enfermedades Metabólicas Asociadas (CIBERDEM), ISCIII, 28029 Madrid, Spain; 9 Pediatrics, Hospital Dr. Josep Trueta, 17007 Girona, Spain; 10 Department of Medical Sciences, University of Girona, 17820 Girona, Spain

**Keywords:** caloric restriction, metabolic programing, DNA methylation, adipose tissue

## Abstract

Limited nutrient supply to the fetus results in physiologic and metabolic adaptations that have unfavorable consequences in the offspring. In a swine animal model, we aimed to study the effects of gestational caloric restriction and early postnatal metformin administration on offspring’s adipose tissue epigenetics and their association with morphometric and metabolic variables. Sows were either underfed (30% restriction of total food) or kept under standard diet during gestation, and piglets were randomly assigned at birth to receive metformin (n = 16 per group) or vehicle treatment (n = 16 per group) throughout lactation. DNA methylation and gene expression were assessed in the retroperitoneal adipose tissue of piglets at weaning. Results showed that gestational caloric restriction had a negative effect on the metabolic profile of the piglets, increased the expression of inflammatory markers in the adipose tissue, and changed the methylation of several genes related to metabolism. Metformin treatment resulted in positive changes in the adipocyte morphology and regulated the methylation of several genes related to atherosclerosis, insulin, and fatty acids signaling pathways. The methylation and gene expression of the differentially methylated *FASN*, *SLC5A10*, *COL5A1*, and *PRKCZ* genes in adipose tissue associated with the metabolic profile in the piglets born to underfed sows. In conclusion, our swine model showed that caloric restriction during pregnancy was associated with impaired inflammatory and DNA methylation markers in the offspring’s adipose tissue that could predispose the offspring to later metabolic abnormalities. Early metformin administration could modulate the size of adipocytes and the DNA methylation changes.

## 1. Introduction

Over the past decades, growing evidence has emerged on the relationship between intrauterine environment and later-life health. Back in the early 90s, Barker et al. showed a relationship between low birth weight and increased susceptibility to metabolic diseases later in life for the first time [1,2]. In 2005, the “Developmental Origins of Health and Disease (DOHaD)” hypothesis, postulated by Gillman et al., stated that limited nutrient supply to the fetus results in physiologic and metabolic adaptations that had unfavorable consequences in the offspring [3,4]. In this sense, several epidemiological studies showed that low calorie intake or malnutrition during pregnancy is associated with increased risk of metabolic and cardiovascular diseases, such as hypertension, insulin resistance, type 2 diabetes, and obesity [1,4,5,6,7,8].

Epigenetics have emerged as one of the mechanisms that could explain the link between the intrauterine environment and later-life consequences [5]. Epigenetic marks are established during development, especially at the embryonic stage, when the entire epigenome is deleted and re-established [7,9]. The influence of environment on the epigenetic marks can have an effect on metabolic tissues (i.e., adipose tissue), altering their lifetime functionality [1,10]. According to the thrifty epigenotype theory, epigenetics play a much more important role in the etiology of such diseases than DNA sequence polymorphisms [11].

Adipose tissue provides thermal protection and somatic support and acts as an endocrine tissue. Its formation starts during the intrauterine period and continues throughout life [12]. Adipose tissue is extremely influenced by environment and fetal nutrition has a critical role in its development [13,14]. Adipose tissue has been recognized as one of the major targets of the fetal programming linking prenatal undernutrition to increased adiposity later in life [15], especially the visceral adipose tissue component [16]. McMillen et al. postulated that scarcity of nutrients during critical periods of development ends in alterations in the structural and functional characteristics of adipose tissue, consequently affecting the endocrine feedback loop between adipose tissue and the system regulating energy balance and body fat mass [15,16].

Metformin is the antidiabetic drug most used worldwide. It works by inhibiting hepatic glucose production and its absorption in the small intestine and increasing glucose peripheral uptake and utilization [17,18,19]. Metformin has multiple sites of action and molecular mechanisms; in this way, it has also been demonstrated its beneficial effects on adolescents with polycystic ovarian syndrome, obesity, and prenatal growth restriction [20,21,22]. Previous data from our group showed that, in a swine animal model, maternal overfeeding during gestation leads to metabolic abnormalities in the offspring, including adipose tissue alterations and that early metformin administration to the offspring, can mitigate these effects [23].

Here, we have used a similar swine animal model to study the effects of gestational caloric restriction and early metformin administration on offspring’s adipose tissue epigenetics and their association with morphometric and metabolic variables.

## 2. Results

### 2.1. Effect of Gestational Caloric Restriction and Metformin Treatment on Offspring Characteristics

**Figure 1:**
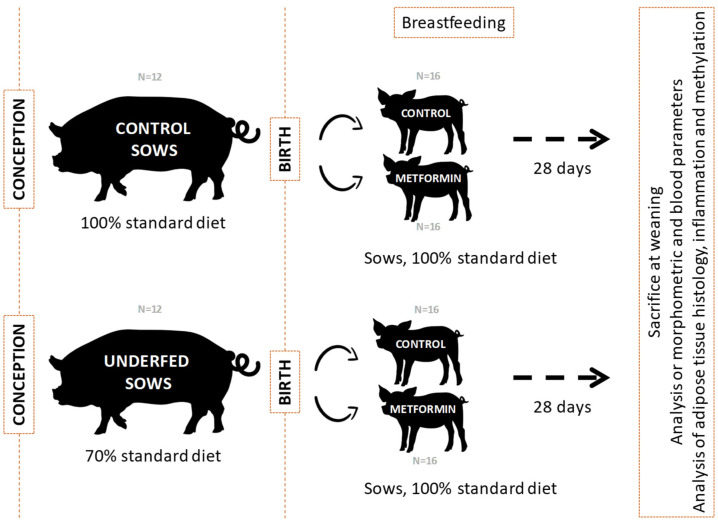
Schematic view of the swine animal model designed for this study.

The schematic view of the swine animal model designed for this study is shown in Figure 1.

Underfed piglets showed no differences compared to control piglets regarding morphometric parameters neither at birth nor at weaning (Table 1). However, when analyzing serum metabolic variables at weaning, underfed piglets showed a partly altered metabolic profile, with higher fructosamine (*p* < 0.001), total cholesterol (*p* = 0.030), CRP (*p* < 0.001), and lower circulating HMW-adiponectin (*p* < 0.001) (Table 1). Moreover, gestational caloric restriction had a slight effect on adipose tissue inflammatory markers as piglets born to underfed sows presented higher expression levels of *TNF-α* and *CCL2* (*p* = 0.017 and *p* = 0.002, respectively) (Table 1).

**Figure 2:**
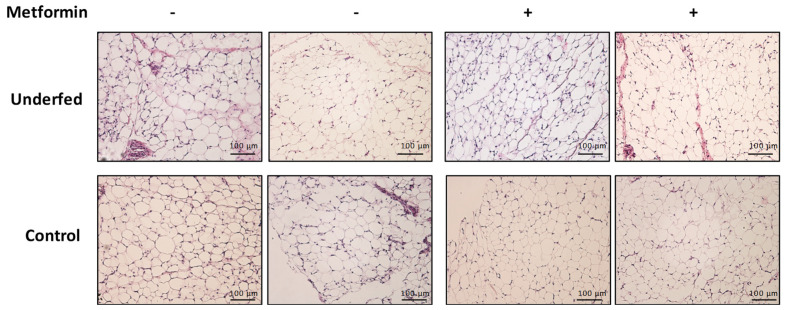
Histology of piglet’s visceral adipose tissue. Hematoxicilin-Eosin staining of retroperitoneal adipose tissue sections from unerfed and control piglets with (+) and without (−) metformin treatment (light microscope, 200×).

Metformin treatment did not trigger statistically significant changes in morphometric parameters of the piglets. However, metformin treatment associated with changes in adipocyte morphology at weaning (Table 1, Figure 2), as adipocytes from piglets treated with metformin showed lower area, perimeter, and diameter compared to placebo (all *p* < 0.01).

### 2.2. Effect of Gestational Caloric Restriction and Metformin Treatment on Adipose Tissue DNA Methylation

**Figure 3:**
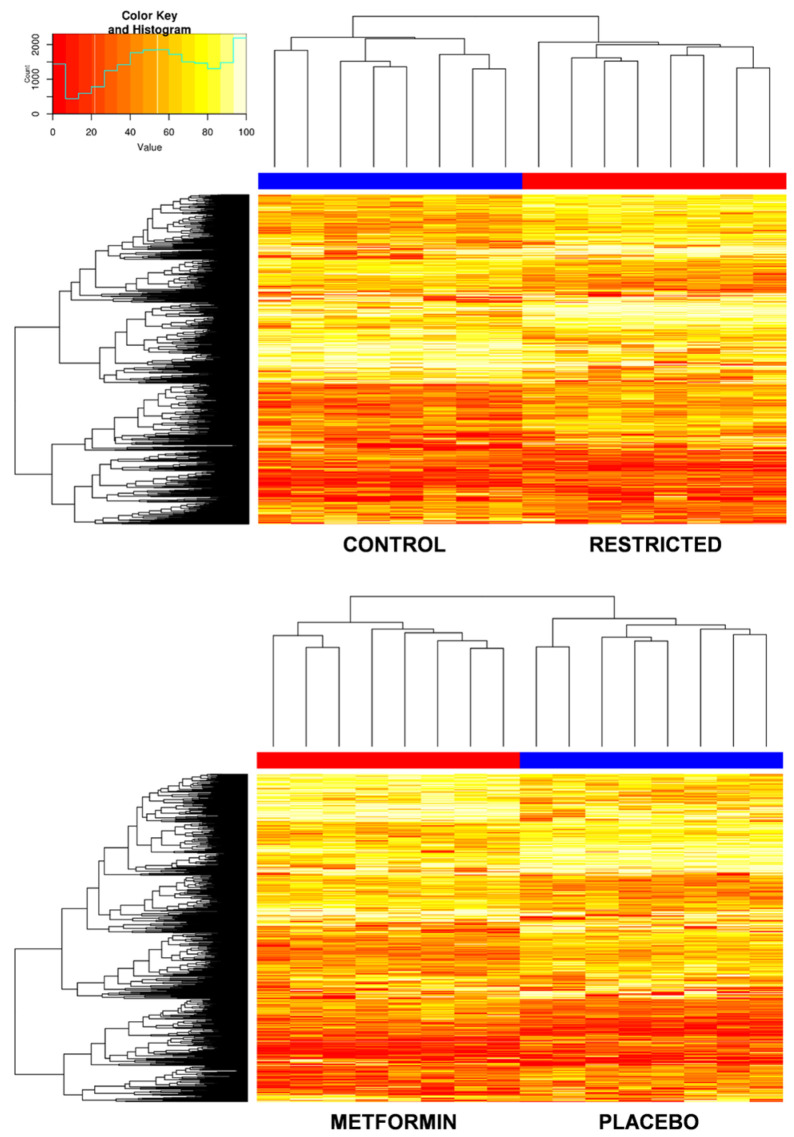
Heat map visualization of the differentially methylated CpG sites in response to restriction or metformin. A scale is shown on the left top, in which red and yellow correspond to a lower and higher methylation status, respectively.

**Figure 4:**
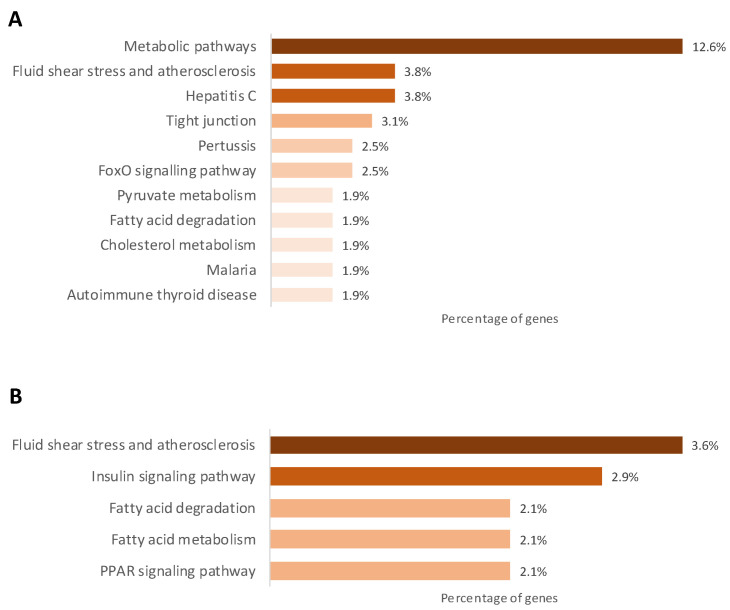
Gene ontology analysis. (**A**) KEGG database pathways of the differentially methylated CPGs with caloric restriction. (**B**) KEGG database pathways of the differentially methylated CpGs with caloric restriction and metformin treatment.

Comparisons between control and underfed offspring at the individual CpGs level revealed 272 CpGs to be differentially methylated. Gestational restriction triggered hypermethylation of 163 CpG and hypomethylation of 109 CpG (Figure 3, Supplemental Table S1). The top five most significant hypermethylated CpGs annotated for the gens *PIWIL1*, *FASN*, *SLC5A10*, *ADAP1,* and *COL5A1*, and the hypomethylated for *NELFB*, *ssc-mir-744*, *ssc-mir-9816*, *CABYR,* and *DKK3*. At the functional level, the list of differentially methylated CpGs was used to identify the most affected signaling pathways. After pathway analysis with DAVID, we found that the enriched signaling pathways of the corresponding genes were mostly involved in pathways related to metabolism (Figure 4A).

Piglets receiving metformin showed methylation differences compared to those receiving placebo in 221 CpGs. The drug hypermethylated 88 CpG sites and hypomethylated 133 CpG sites (Figure 3, Supplemental Table S2). The most significant CpGs hypermethylated annotated for *HTT*, *NELFB*, *ALOX5AP*, *TSPO,* and ssc-mir-9814, and the hypomethylated for *PIWIL1*, *INPPL1*, *ALG10*, *PRLHR,* and *IL10*. The corresponding significant enriched signaling pathways were atherosclerosis, insulin signaling, and fatty acids metabolism (Figure 4B).

### 2.3. Gestational Caloric Restriction Regulates DNA Methylation and Gene Expression in Adipose Tissue

**Figure 5:**
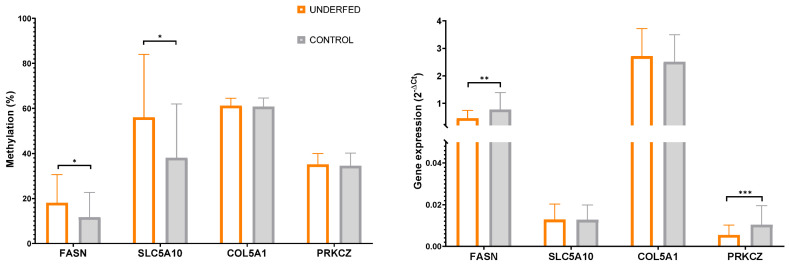
Graphs comparing the DNA methylation and gene expression levels of *FASN, SLC5A10, COL5A1,* and *PRKCZ* in adipose tissue of underfed and control piglets (* *p* < 0.05, ** *p* < 0.01 and *** *p* < 0.001).

Pyrosequencing analysis in all adipose tissue samples showed that *FASN* was affected by gestational caloric restriction at both methylation and gene expression levels in opposite directions, as underfed piglets showed higher *FASN* methylation levels (*p* = 0.043) and lower *FASN* gene expression levels (*p* = 0.018) compared to control. *SLC5A10* methylation levels were higher in the underfed group (*p* = 0.032) compared to control but no differences were observed in *SLC5A10* gene expression. Gestational caloric restriction had no effect on *COL5A1* methylation and gene expression. Gestational caloric restriction did not affect *PRKCZ* methylation, but *PRKCZ* gene expression levels were lower in underfed (*p* = 0.003) compared to controls piglets (Table 2 and Figure 5). No significant differences were observed with metformin treatment in any of the studied genes.

### 2.4. DNA Methylation and Gene Expression in Adipose Tissue Associated with the Metabolic Profile of the Piglets Born to Underfed Sows

**Figure 6:**
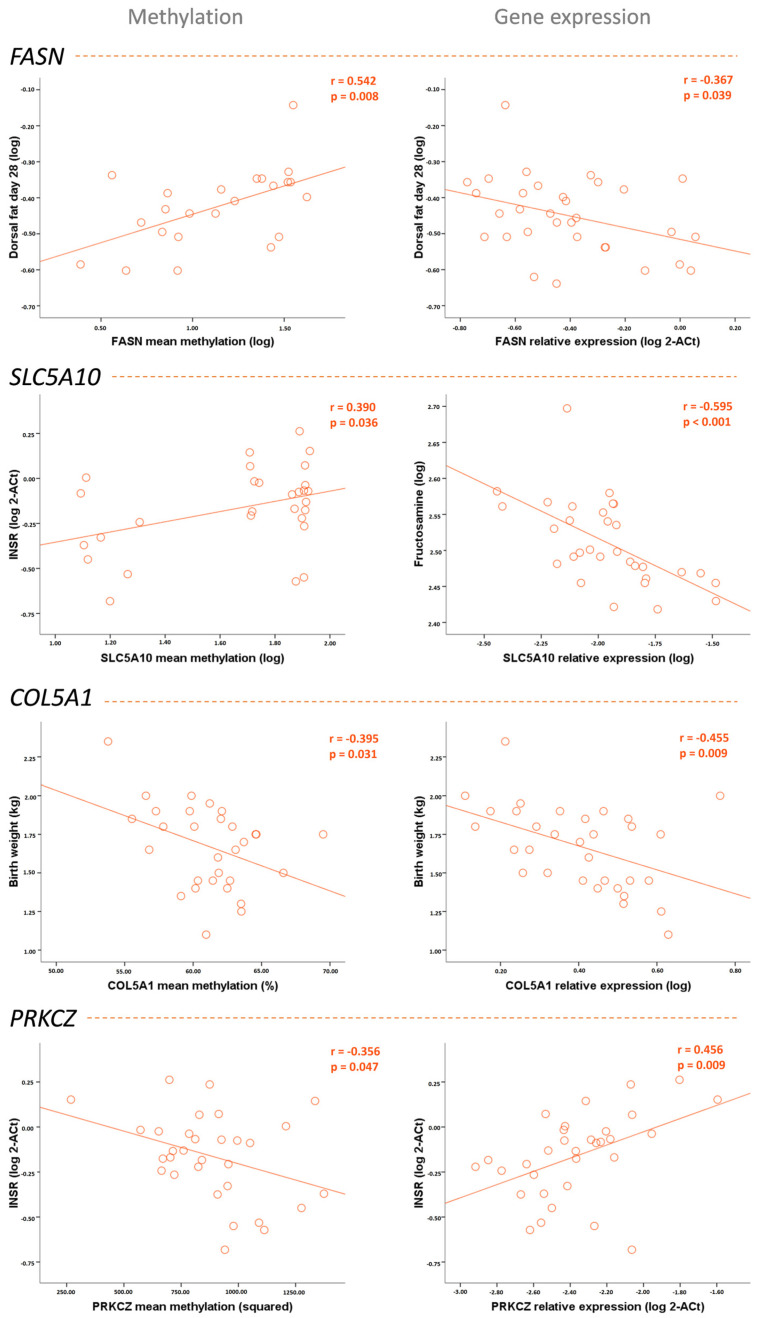
Correlation graphs showing the association between DNA methylation and gene expression of the studied genes (*FASN*, *SLC5A10*, *COL5A1*, and *PRKCZ*) and several metabolic parameters in underfed piglets.

In piglets born to underfed, but not control, sows, *FASN* methylation associated with an unfavorable metabolic profile with higher weight, weight gain since birth, abdominal circumference, and dorsal fat thickness and *FASN* expression showed opposite associations (lower dorsal fat thickness) (all *p* < 0.05). Both *SLC5A10* methylation and expression associated with a favorable metabolic profile. *SLC5A10* methylation associated with lower birth weight (*p* < 0.01) and higher HDL-cholesterol and *INSR* expression (both *p* < 0.05), while *SLC5A10* gene expression associated with lower weight (*p* < 0.05), weight gain since birth (*p* < 0.02), dorsal fat thickness (*p* < 0.001), and fructosamine (*p* < 0.001), and higher HMW adiponectin (*p* < 0.02). *COL5A1* also associated with a favorable metabolic profile. *COL5A1* methylation associated with lower birth weight (*p* < 0.05) and higher HDL-cholesterol and *INSR* expression (all *p* < 0.05), while *COL5A1* gene expression associated with lower birth weight (*p* < 0.02), weight (*p* < 0.05), weight gain since birth (*p* < 0.05), and fructosamine (*p* < 0.05) and higher HDL-cholesterol (*p* < 0.001). *PRKCZ* methylation associated with an unfavorable metabolic profile with lower HDL, *INSR,* and *IRS1* expression (all *p* < 0.05), while *PRKCZ* gene showed opposite associations with higher HDL-cholesterol (*p* < 0.05) and *INSR* expression (*p* < 0.02) (Table 3 and Figure 6).

## 3. Discussion

Our work studied the effect of gestational caloric restriction and early metformin administration on the offspring’s adipose tissue DNA methylome in a pig model for the first time, and showed that caloric restriction during pregnancy regulates DNA methylation and gene expression in the offspring’s adipose tissue that, in turn, associated with the metabolic profile of the offspring.

Our model of fetal undernutrition shows differences in the metabolic and inflammatory status of the piglets. Piglets born to underfed sows showed a worse metabolic profile, with higher fructosamine, total cholesterol, C-reactive protein, and lower HMW-adiponectin. These results show alterations in glucose, lipid, and inflammation levels, all of them markers of metabolic syndrome. Adipose tissue inflammatory markers were also altered by undernutrition, which led to higher expression levels of the pro-inflammatory genes *TNF-α* and *CCL2*. In this sense, previous findings in the literature stated that adipose tissue adjusts its function and metabolism in response to the nutritional status [24]. All these results are in line with previous studies highlighting the link between gestational undernutrition and the likelihood of developing cardiometabolic abnormalities later in life [25,26] and post-natal inflammation [5,27,28,29,30].

On the other hand, metformin treatment had an impact on visceral adipose tissue, reducing the adipocyte area, perimeter, and diameter, as we previously described in a similar swine animal model of gestational overfeeding [23]. Metformin regulates adipogenesis [31], fibrosis [32,33], and fatty acids metabolism [34] of the adipose tissue. The beneficial effects of lactation metformin treatment on offspring adipose tissue had also been described in mice [35].

The methylation array showed hypermethylation of 163 CpGs and hypomethylation of 109 CpGs associated with gestational caloric restriction. Most of the CpGs annotated for genes related to metabolism, showing the adaptation of the piglet’s metabolism in response to the caloric-restricted environment during pregnancy. It is known that caloric restriction at any life period will have metabolic effects on the body [36,37,38]. Metformin, in turn, hypermethylated 88 CpGs and hypomethylated 133 CpGs, a great deal of them being related to insulin signaling and fatty acid metabolism. These results are plausably related to the metformin mechanism of action since both pathways are affected by this drug [39].

*FASN* was hypermethylated by gestational caloric restriction (higher *FASN* methylation in the underfed group) and triggered opposite effects on gene expression, showing lower *FASN* expression in the underfed group. Moreover, in underfed piglets, *FASN* methylation associated with higher weight and dorsal fat. FASN is known to play a central role in de novo lipogenesis in mammals, catalyzing the reaction in the synthesis of palmitate from acetyl-CoA and malonyl-CoA [40]. To our knowledge, there are no previous studies investigating the effect of gestational caloric restriction on *FASN* methylation in the adipose tissue. Nevertheless, *FASN* gene expression has been broadly related to the regulation of body weight and the development of obesity [41].

Diet-induced hypermethylation of *SLC5A10* in the underfed group, whereas no effect on gene expression was observed. SLC5A10 encodes for a sodium-dependent sugar transporter; in particular, SLC5A10 is known to transport mannose, fructose [42,43], and a monosaccharide similar to glucose, the 1.5-anhudroglucitol [44]. In humans, it is thought to be exclusively expressed in the kidney [42]. In the underfed group, negative associations of gene expression with weight, fat, and fructosamine parameters were observed. This could be explained by its role in fructose absorption, considering that fructose levels have been associated with insulin-resistance and liver lipogenesis [43].

*COL5A1* encodes for the alpha 1 chain of the collagen V protein, and positively correlates with adipose tissue expansion [45]. Contrary to previous bibliography, we acknowledge a negative correlation between morphometric parameters and *COL5A1* gene expression. However, Dankel et al. found an upregulation of COL5A1 after major weight loss following bariatric surgery, proposing a switch to metabolically favorable remodeling of adipose tissue after profound fat loss [46]. In our experimental model, this inverse correlation could compensate for the adverse environment of nutrition during pregnancy.

Undernutrition diminished *PRKCZ* gene expression in adipose tissue. Interesting associations were observed in the underfed piglets in both methylation and gene expression. Methylation of *PRKCZ* in the underfed group negatively correlated with *INSR* and *IRS1* expression, and positively with CRP, showing a relationship between methylation and inflammatory and insulin resistance markers. This relationship was inversely observed in part with gene expression since it positively correlated with *INSR* expression. PRKCZ transmits insulin signaling downstream, through its interaction with IRS1 [47]. Due to its function, PRKCZ is an important factor in the pathogenesis of type 2 diabetes (T2D) [48]. Our results reinforce previous literature on the relationship between PRKCZ methylation and the initiation and progression of T2D [48].

We acknowledge that our swine model may have some limitations as we did not observe differences in the offspring morphometric variables nor alterations in the adipose tissue histology, as has been previously described in different animal species. In sheep, a 50% restriction of their nutritional requirements both during early [49] and late [29] pregnancy reduced the body weight of the offspring at birth compared to control dams (which received 100% of requirements) and showed lower subcutaneous fat deposition. In rats with a 50% maternal food-restriction during pregnancy, the pups showed lower body weight at birth and at PD22 and PD42 compared to control pups (with ad libitum maternal diet) and higher retroperitoneal adipose tissue and visceral fat [50,51]. Our model of gestational caloric restriction was just about 30% during pregnancy, while in resembling studies caloric intake was reduced up to 50% during the third trimester [52]. However, the differential impact of fetal malnutrition may be related to the amount and timing of the malnutrition exposure [53] and to the ability of adipogenic precursors to develop in response to different nutritional exposures [54]. Moreover, it is known that the pig under low calorie intake is able to mobilize maternal energy reserves to support placental and fetal development. If the sow is healthy, a modest reduction in the dietary intake of energy is not enough to cause growth defects in the piglets [9]. Another limitation is the limited genome information in the pig species. Nevertheless, pigs have proven to be a valuable animal model in nutritional, metabolic, and cardiovascular research and in some other biomedical research areas (toxicology, neurobiology). The main reasons to select this animal for research is the large resemblance with human in regard to their (neuro)anatomy, the gastro-intestinal tract, body size, body composition, and the omnivorous food choice and appetite. Moreover, both humans and pigs are prone to the development of obesity and related cardiovascular diseases such as hypertension and atherosclerosis [11,55,56]. Pig models fill the gap between rodent models and primate species including humans and such studies are essential to understand the link between prenatal environment and postnatal development. From this standpoint, our research also reinforces the idea of epigenetics explaining, at least in part, such links between in utero environment and offspring health later in life. Further work needs to be completed to elucidate all the components involved in such links, to be able to early detect and prevent long-term cardiometabolic diseases. Moreover, it would be interesting to study the effect of other antidiabetic drugs to validate our results and substantiate the argument presented. Our data fail to show the beneficial effects of metformin administration on the metabolic profile of the underfed piglets that was observed in our previous swine model of gestational overfeeding [23]. This may be related to the lack of morphological alterations observed in the offspring of caloric restricted sows.

In conclusion, our swine model showed that caloric restriction during pregnancy was associated with impaired inflammatory and DNA methylation markers in the offspring’s adipose tissue, which could predispose the offspring to later metabolic abnormalities. Early metformin administration could modulate the size of adipocytes and the DNA methylation changes.

## 4. Materials and Methods

### 4.1. Animal Model and Samples

**Figure 7:**
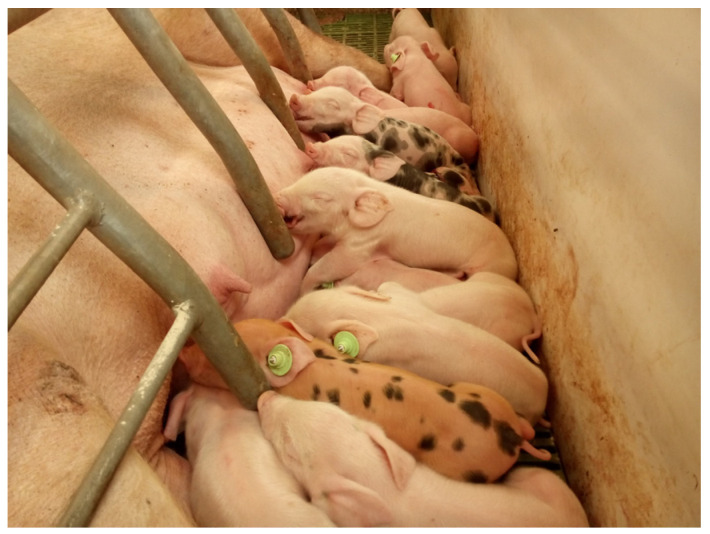
Image of a sow nursing its piglets.

A model of gestational caloric restriction was developed using sows obtained from the crossbreed between Landrace x Duroc sows and Pietrain purebred boars. The study was approved by the Ethical Committee of Institut de Recerca i Tecnologia Agroalimentàries (IRTA) and animal housing and experimentation was performed following national and institutional guidelines for Good Experimental Practices. Pregnant sows were separated into two dietary groups: (1) control (n = 12), which were fed with a standard diet (2050 Kcal/kg feed; 5% fat, 15% crude protein, 48% carbohydrate plus sugars, 7% crude fiber); and (2) underfed (n = 12), which were fed with 70% of the standard diet. Diet intervention started at conception and continued during the pregnancy. Diet intervention finished at birth and during lactation sows from both groups were fed with a standard diet (Figure 7). At birth, piglets born to control (n = 32, 16 males and 16 females) and underfed (n = 32, 16 males and 16 females) were randomly divided into two different treatments: (1) metformin (50 mg/kg/day prepared in water with corn starch 4 g/day; n = 16, 8 males and 8 females) and (2) vehicle alone (water with corn starch 4 g/day; n = 16, 8 males and 8 females), which were administered orally. The dose of metformin was constant per kg of the animal throughout all the experiment and similar to the one used by Gonzalez-Bulnes et al. [57] (approximately 45 mg/kg/day). Piglets were sacrificed at weaning (28 days old). On the same day, after a fasting period, blood was drawn, clotted at room temperature for 30 min, and centrifuged at 2000× *g* for 15 min to obtain serum. Adipose tissue samples from the visceral fat layer at retroperitoneal level were collected at sacrifice.

### 4.2. Morphometry

Piglet’s weight was obtained at birth and sacrifice using a universal scale. Abdominal circumference was measured at the level of the last rib with a metric tape. Subcutaneous dorsal fat thickness was assessed using a calibrated caliper [58].

### 4.3. Metabolic Markers

Serum determinations of metabolic markers including glucose, insulin, fructosamine, HDL and total cholesterol, triglycerides, C-reactive protein (CRP), and High Molecular Weight (HMW) adiponectin were performed using standardized tests as previously described [23]. Homeostatic model assessment of insulin resistance (HOMA-IR) and HOMA-β indexes were calculated as previously described [59].

### 4.4. Adipose Tissue Histology

Histological sections of retroperitoneal adipose tissue were obtained and the morphometric studies to quantify the area, perimeter, and diameter of the adipocytes were performed as previously described [23].

### 4.5. Whole Methylome Analysis

A whole methylome analysis was performed in 32 samples of retroperitoneal adipose tissue [16 control (8 with metformin treatment and 8 with vehicle) and 16 underfed (8 with metformin treatment and 8 with vehicle)] using RRBS (Reduced Representation Bisulfite Sequencing) by Diagenode (Seraing, Belgium) as previously described [23]. Briefly, high-quality genomic DNA from eight randomly selected samples of adipose tissue per diet and treatment was isolated and bisulfite converted using GentraPureGene tissue kit (Qiagen, Hilden, Germany) and EZ DNA Methylation-Gold Kit (Zymo Research, Irvine, CA, USA), respectively. Sequencing was conducted on a HiSeq 3000 (Illumina, San Diego, CA, USA). The swine reference genome susScr3, from the USCS Genome Browser, was used for alignment and methylation calling. Raw data are available under GEO accession number GSE169515. Differentially methylated cytosines in CpG cytosine and guanine dinucleotides sites (CpGs) (at least 15% of methylation difference and adjusted *p*-value < 0.05) and their annotated genes were obtained.

Several CpGs and their annotating genes were selected according to their significance (lower *p*-value) and their relevance (presence of two or more differentially methylated CpGs annotating for the same gene within 150 pb of distance) to be studied in all adipose tissue samples (Supplemental Table S3). *FASN* had 11 CpGs, with a mean methylation difference between groups of 24%. *SLC5A10* presented 4 CpG differentially methylated and a mean methylation difference of 22%. *COL5A1* had 2 CpGs with a difference of methylation of 20%. *PRKCZ* was also chosen for validation since metformin had the opposite effect as diet on CpGs methylation. Restriction hypermethylated 2 CpGs annotating for *PRKCZ*, with a mean differential methylation of 19%, and metformin also hypomethylated 2 CpGs with a differential mean methylation of −17%. *FASN* methylation was also inversely affected by metformin, while diet hypermethylated 11 CpGs, metformin hypomethylated 10 CpGs.

### 4.6. Pyrosequencing Analysis

DNA methylation of the selected CpGs was analyzed in all adipose tissue samples (n = 16 samples per diet and treatment intervention) by pyrosequencing as previously described [23]. Briefly, bisulphite DNA was amplified by PCR and pyrosequenced in a PyroMark Q96 ID (Qiagen, Germany) using specific primers (Supplementary Table S3). DNA methylation levels for each gene were calculated as the average of all the differentially methylated CpGs with the same gene annotation.

### 4.7. Gene Expression Analysis

The expression of several adipose tissue genes related to inflammation as well as the genes annotating for the selected CpGs was analyzed in all adipose tissue samples (n = 16 samples per diet and treatment intervention) by RT-PCR as previously described [23]. Briefly, total RNA was extracted using the RNeasy Mini Kit and retrotranscribed to cDNA with the High-Capacity Reverse Transcription Kit (Thermo Fisher Scientific, Waltham, MA, USA). The expression of the genes related to inflammation was tested with the following TaqMan Gene Expression Assays from Thermo Fisher Scientific: *TNFA* (Ss03391318_g1), *IL6* (Ss03384604_u1), *CCL2* (Ss03394377_m1), *INSR* (Ss03375405_u1), and *IRS1* (Ss04327584_m1). The expression of the genes selected from methylation studies was tested with the following TaqMan Gene Expression Assays: *FASN* (Ssc03386194_u1), *PRKCZ* (Ssc04321729_m1), *SLC5A10* (Ss03379037_u1), and *COL5A1* (Ssc03379198_u1). *GAPDH* (Ss03374854_g1) was used as a housekeeping control. Relative expression levels were calculated according to the 2^−ΔCt^ method.

### 4.8. Statistics and Gene Ontology Analyses

Statistical analyses were completed with the program SPSS 22.0 (IBM, Armonk, NY, USA). To improve symmetry, non-normally distributed variables were log-transformed. Data are shown as mean ± standard deviation (SD). The comparisons between maternal diet and treatment interventions were assessed using Two-way ANOVA tests. Pearson correlation was used to analyze associations between variables. Combined data for males and females are shown since no sex differences were evident. Significance was set at *p* < 0.05. Gene ontology analysis was performed on the website DAVID bioinformatics [60], pathways come from the KEGG database, with a FDR cut-off of 10.

## Figures and Tables

**Table 1:** Morphometric, metabolic variables, and adipose tissue characteristics of the studied piglets at weaning.

	Control		Underfed		Two-Way ANOVA for Interaction	Gestational Caloric Restriction	Metformin Treatment
	Vehicle N = 16	Metformin N = 16	Vehicle N = 16	Metformin N = 16			
**Morphometry**					***p*-value**	***p*-value**	***p*-value**
Birth weight (Kg)	1.67 ± 0.3		1.68 ± 0.2		-	0.890	-
Weight (kg)	7.91 ± 1.3	7.06± 1.3	7.12± 1.4	7.46 ± 1.6	0.093	0.572	0.477
Weight gain since birth (kg)	6.24 ± 1.3	5.39± 1.3	5.49± 1.3	5.81 ± 1.4	0.082	0.636	0.440
Abdominal circumference (cm)	34.59 ± 2.7	32.93 ± 3.2	33.06± 2.3	33.94 ± 2.6	0.064	0.610	0.636
Dorsal fat thickness (cm)	0.38 ± 0.12	0.37 ± 0.10	0.36± 0.07	0.37 ± 0.12	0.781	0.887	0.732
**Metabolic**							
Glucose (mg/dL)	301.06 ± 123.1	332.56± 147.8	314.19± 86.3	282.13 ± 88.9	0.271	0.518	0.992
Insulin (mU/L)	0.87 ± 0.1	0.85 ± 0.1	0.89 ± 0.1	0.84 ± 0.1	0.550	0.904	0.195
HOMA-IR	0.64 ± 0.3	0.70 ± 0.3	0.70 ± 0.3	0.59 ± 0.2	0.266	0.994	0.562
Fructosamine (µM)	296.81 ± 25.0	271.50 ± 19.9	329.47 ± 60.0	324.14 ± 34.8	0.163	**<0.001**	0.094
Total cholesterol (mg/dL)	189.00 ± 44.8	167.75 ± 46.6	211.31 ± 92.5	227.50 ± 70.8	0.152	**0.030**	0.869
HDL-cholesterol (mg/dL)	63.81 ± 10.8	58.63 ± 7.0	60.00 ± 13.8	64.69 ± 14.3	0.101	0.709	0.934
Triglycerides (mg/dL)	100.63 ± 40.4	116.25 ± 35.7	97.81 ± 31.4	108.69 ± 49.8	0.561	0.670	0.169
C-reactive protein (mg/L)	0.007 ± 0.002	0.007 ± 0.002	0.011 ± 0.003	0.012 ± 0.004	0.753	**<0.001**	0.731
HMW adiponectin (mg/L)	1.07 ± 0.2	1.06 ± 0.2	0.88 ± 0.3	0.85 ± 0.1	0.921	**<0.001**	0.691
**Adipose Tissue**							
Adipocyte area (µm)	1316.56 ± 267.1	1109.00 ± 365.1	1273.63 ± 258.8	1074.94 ± 282.4	0.952	0.602	**0.008**
Adipocyte perimeter (µm)	135.88 ± 14.9	123.81 ± 22.3	134.69 ± 13.2	123.69 ± 17.3	0.903	0.870	**0.009**
Adipocyte diameter (µm)	37.69 ± 5.1	33.69 ± 5.3	37.50 ± 4.1	34.44 ± 5.3	0.707	0.820	**0.006**
*TNF-* *α expression*	0.006 ± 0.004	0.005 ± 0.003	0.007 ± 0.005	0.016 ± 0.024	0.132	**0.017**	0.277
*IL6 expression*	0.010 ± 0.010	0.009 ± 0.007	0.011 ± 0.009	0.010 ± 0.006	0.523	0.434	0.777
*CCL2 expression*	0.23 ± 0.1	0.29 ± 0.2	0.42 ± 0.3	0.45 ± 0.3	0.792	**0.002**	0.424
*INSR expression*	0.62 ± 0.3	0.66 ± 0.3	0.71 ± 0.4	0.87 ± 0.4	0.503	0.198	0.259
*IRS1 expression*	0.37 ± 0.2	0.34 ± 0.2	0.41 ± 0.2	0.44 ± 0.2	0.573	0.108	0.947

Data are shown as mean ± SD. In bold statistical significant *p*-values.

**Table 2:** DNA methylation and gene expression in adipose tissue from the piglets at weaning.

	Control		Underfed		Two-Way ANOVA for Interaction	Gestational Caloric Restriction	Metformin
	Vehicle N = 16	Metformin N = 16	Vehicle N = 16	Metformin N = 16		*p*-Value	*p*-Value
**Methylation**							
FASN	11.95± 10.9	11.50 ± 11.5	20.57 ± 13.8	15.43 ± 10.8	0.712	**0.043**	0.586
SLC5A10	37.51 ± 17.8	38.67 ± 29.9	55.73 ± 27.9	56.35 ± 29.1	0.652	**0.032**	0.575
COL5A1	61.16 ± 4.2	60.44 ± 3.4	60.97 ± 2.8	61.41 ± 3.9	0.536	0.685	0.892
PRKCZ	28.02 ± 5.9	30.04 ± 2.9	29.07 ± 3.2	29.87 ± 4.9	0.795	0.727	0.209
**Gene Expression**							
FASN	0.69 ± 0.7	0.87 ± 0.6	0.47 ± 0.3	0.45 ± 0.3	0.319	**0.018**	0.396
SLC5A10	0.012 ± 0.007	0.013 ± 0.007	0.013 ± 0.007	0.013 ± 0.007	0.663	0.861	0.821
COL5A1	2.46 ± 1.1	2.57 ± 0.9	2.90 ± 1.2	2.53 ± 0.8	0.323	0.401	0.861
PRKCZ	0.010 ± 0.009	0.011 ± 0.009	0.005 ± 0.003	0.006 ± 0.006	0.864	**0.003**	0.439

Data are shown as mean ± SD. In bold statistical significant *p*-values.

**Table 3:** Pearson correlation coefficients between DNA methylation and gene expression in adipose tissue and morphometric and metabolic variables in underfed piglets at weaning.

Underfed								
	*FASN* Methylation	*FASN* Gene Expression	*SLC5A10* Methylation	*SLC5A10* Gene Expression	*COL5A1* Methylation	*COL5A1* Gene Expression	*PRKCZ* Methylation	*PRKCZ* Gene Expression
**Morphometry**								
Birth weight	−0.034	−0.150	−0.563 **	−0.153	−0.395 *	−0.455 **	0.266	−0.029
Weight	0.528 *	−0.260	−0.320	−0.423 *	−0.203	−0.435 *	0.036	0.005
Weight gain since birth	0.588 **	−0.267	−0.217	−0.459 **	−0.147	−0.388 *	0.001	0.012
Abdominal circumference	0.447 *	−0.254	−0.026	−0.257	−0.192	−0.167	−0.075	0.055
Dorsal fat thickness	0.542 **	−0.367 *	−0.001	−0.558 ***	−0.208	−0.337	−0.090	−0.201
**Metabolic Markers**								
Glucose	0.250	−0.220	0.052	−0.309	−0.002	0.104	0.024	0.016
Insulin	−0.227	0.006	−0.213	−0.115	−0.135	−0.026	−0.055	−0.012
HOMA-IR	0.124	−0.201	−0.052	−0.304	−0.067	0.036	0.043	−0.036
Fructosamine	0.215	−0.218	−0.037	−0.595 ***	−0.274	−0.374 *	−0.007	−0.315
Total cholesterol	0.199	0.154	0.289	−0.192	0.414*	0.103	−0.223	0.337
HDL-cholesterol	0.141	0.242	0.384 *	0.222	0.388*	0.634 ***	−0.432 *	0.420 *
Triglycerides	−0.208	−0.149	0.204	0.184	−0.173	−0.347	−0.134	0.065
C-reactive protein	0.017	0.055	−0.270	−0.276	−0.341	−0.337	0.424 *	−0.335
HMW adiponectin	−0.123	0.267	−0.149	0.463 **	0.080	0.170	−0.147	0.264
**Gene Expression in the Adipose Tissue**								
*TNFA expression*	−0.028	−0.014	0.061	−0.088	0.329	0.122	−0.063	0.288
*IL6 expression*	−0.147	−0.015	0.073	−0.209	0.102	0.075	−0.201	0.052
*CCL2 expression*	−0.149	0.177	−0.028	0.109	0.036	0.240	−0.335	0.267
*INSR expression*	−0.261	0.043	0.390 *	0.077	0.454 *	0.291	−0.356 *	0.456 **
*IRS1 expression*	−0.249	0.002	0.213	0.097	0.333	0.355 *	−0.378 *	0.264

* *p* < 0.05, ** *p* < 0.01, *** *p* < 0.001.

## Data Availability

The datasets generated and analyzed during the current study are available from the corresponding author on reasonable request. Raw data from the DNA methylome are available under GEO accession number GSE169515.

## Supplementary Materials

The following supporting information can be downloaded at: https://www.mdpi.com/article/10.3390/ijms25021128/s1.
