# Supplementary material for: Gestational Caloric Restriction Alters Adipose Tissue Methylome and Offspring’s Metabolic Profile in a Swine Model
Source: Int J Mol Sci. 2024 Jan 17;25(2):1128. doi: 10.3390/ijms25021128 (PMC10816194; doi:10.3390/ijms25021128)
Supplement: Supplementary file 1 [file ijms-25-01128-s001.zip › Supplemental Table S3_primers and annealing temperatures.pdf]

Supplemental Table S3. Sequence of the primers used and their annealing temperatures.

Array information			Primers for the validation			
CpGs location	p-value	Gene annotation	Primer	Sequence	Amplicon length	Annealing temperature
chr12:909812	9.90 $x10^{-5}$	***FASN***	Fw	GAGTTTGTTGGAGGAAAGTTTTT	505	51ºC
chr12:909822	1.55 $x10^{-4}$		Rv	BIOT-CTCCTCTAAAAACAACAAAATCCC		
chr12:909834	4.64 $x10^{-7}$		Seq	GAGGTTTTGTGTTGGGAA		
chr12:909841	1.08 $x10^{-4}$					
chr12:909977	3.06 $x10^{-12}$					
chr12:909979	5.43 $x10^{-13}$					
chr12:909982	3.73 $x10^{-11}$					
chr12:909994	1.60 $x10^{-11}$					
chr12:910001	7.24 $x10^{-11}$					
chr12:910003	8.87 $x10^{-12}$					
chr12:910010	1.78 $x10^{-12}$					
chr12:62731379	2.54 $x10^{-5}$	***SLC5A10***	Fw	AGGGAGTTAGGTTTTTAGAAAGGTT	402	57ºC
chr12:62731390	2.21 $x10^{-8}$		Rv	BIOT-AAAAATCCCTCACTCACTCATAATT		
chr12:62731408	2.15 $x10^{-12}$		Seq	TTATTTGATAATTTATTA		
chr12:62731521	1.20 $x10^{-6}$		Seq	GTTTTTAATAAAGTTGAA		
chr1:307679069	8.92 $x10^{-5}$	***COL5A1***	Fw	GGTTTTTGGGTTAGTAGGATAGAGTAGA	206	56ºC
chr1:307679087	1.38 $x10^{-6}$		Rv	BIOT-AAACCCCAACCCCTACCCATC		
			Seq	GGGGATTAGGGTTTTG		
chr6:58302486	1.33 $x10^{-3}$	***PRKCZ***	Fw	TGAGGATTTTTAGTATTTGGTGTG	267	56ºC
chr6:58302508	4.95 $x10^{-4}$		Rv	BIOT-CTAAAAACCAACTCCAACAC		
			Seq	GGGTTTTTGAGATTA		
